# Improved Detection of Visual Field Progression Using a Spatiotemporal Boundary Detection Method

**DOI:** 10.1038/s41598-018-37127-z

**Published:** 2019-03-15

**Authors:** Samuel I. Berchuck, Jean-Claude Mwanza, Angelo P. Tanna, Donald L. Budenz, Joshua L. Warren

**Affiliations:** 1 Department of Statistical Science and Forge, Duke University, Durham, NC USA; 2 Department of Ophthalmology, University of North Carolina-Chapel Hill, Chapel Hill, NC USA; 3 Department of Ophthalmology, Northwestern University, Illinois, USA; 4 Department of Biostatistics, Yale University, Connecticut, USA

## Abstract

Glaucoma is the leading cause of irreversible blindness worldwide and requires regular monitoring upon diagnosis to ascertain whether the disease is stable or progressing. However, making this determination remains a difficult clinical task. Recently, a novel spatiotemporal boundary detection predictor of glaucomatous visual field (VF) progression (STBound) was developed. In this work, we explore the ability of STBound to differentiate progressing and non-progressing glaucoma patients in comparison to existing methods. STBound, Spatial PROGgression, and traditional trend-based progression methods (global index (GI) regression, mean regression slope, point-wise linear regression, permutation of pointwise linear regression) were applied to longitudinal VF data from 191 eyes of 91 glaucoma patients. The ability of each method to identify progression was compared using Akaike information criterion (AIC), full/partial area under the receiver operating characteristic curve (AUC/pAUC), sensitivity, and specificity. STBound offered improved diagnostic ability (AIC: 197.77 vs. 204.11–217.55; AUC: 0.74 vs. 0.63–0.70) and showed no correlation (r: −0.01–0.11; p-values: 0.11–0.93) with the competing methods. STBound combined with GI (the top performing competitor) provided improved performance over all individual metrics and compared to all metrics combined with GI (all p-values < 0.05). STBound may be a valuable diagnostic tool and can be used in conjunction with existing methods.

## Introduction

The chronic and progressive nature of open-angle glaucoma (OAG) requires patients to be monitored over time in order to establish whether the disease is progressing and at what rate. Determination of progression status is important for timely adjustment or escalation of treatment. However, detecting glaucomatous functional progression is challenging^1–5^. Despite the use of visual field (VF) data for clinical decision making and as an endpoint in clinical trials^5–10^, there is no widely accepted reference standard for determining VF progression. One of the factors contributing substantially to this difficulty is the variability in test results. This variability is driven by patient-related factors such as inattention as well as disease-associated factors. Glaucomatous optic nerve damage is in and of itself associated with increased fluctuation in visual sensitivity. This intra-test and inter-test variability, can mimic VF worsening (or progression) in the absence of true disease worsening or mask a true glaucoma-related functional change.

Standard achromatic perimetry (SAP) is the most commonly used functional testing method for VF assessment. SAP, which can be performed with the Humphrey Field Analyzer (HFA, Carl Zeiss Meditec, Dublin, CA, USA), the Octopus Perimeter (Haag-Streit, Koniz, Switzerland), or other similar devices, provides sensitivity estimates at each test point in the VF. These sensitivities are monitored over time to determine if glaucomatous progression is occurring for an eye. For decision-making about progression status, most clinicians use either their own subjective assessment of VF series or assessment of statistically significant progression determined by software with automated algorithms built into SAP devices.

Currently, statistical modeling for assessing VF progression mainly relies on event- or trend-based analyses^11^. Event-based analyses (e.g., HFA Guided Progression Analysis (GPA)) express progression status in a binary manner (progression or no progression) after comparing a recent VF test with baseline results, often without considering test results from all other visits^12^. These methods use pre-specified levels of deterioration in order to define progression. Trend-based analyses (e.g., HFA-compatible PROGRESSOR, Medisoft Inc, Leeds, UK) assess VF progression using rates of loss determined by regression modeling of global indices (e.g., mean deviation)^13–15^, sectoral mean sensitivity, and/or sensitivity of individual test points over time^16^. Advanced methods including spatial filters^11,17,18^, statistical modeling of spatial correlation^19–21^, general hierarchical Bayesian modeling^22^, and machine learning^23^ have also been introduced to properly differentiate true signal from non-specific variability.

The majority of previous models in this setting focus on changes in VF sensitivities over time in some form (e.g., changes from baseline; trends over time), but do not explicitly consider how changes in variability, and more specifically spatial correlation, may be an equally important indicator of progression. This idea is partially supported by the known inverse relationship between VF sensitivities and testing variability^24^. We previously developed statistical theory for a novel spatiotemporal boundary detection model that considers the anatomical relationship between the retinal nerve fiber layer and the optic nerve when modeling longitudinal VF data, and successfully quantifies changes in spatial correlation across time. This measure of variability, hereafter referred to as STBound, was shown to be an important predictor of glaucomatous VF progression^25^. The purpose of this study is to extend STBound and validate it through comparison with existing VF progression statistical methods. Classification accuracy and the speed of the competing methods to correctly diagnose progression are both investigated.

## Methods

### Data description

We source data from the Vein Pulsation Study Trial in Glaucoma and the Lions Eye Institute trial registry, Perth, Western Australia, which contain 1,448 VFs from 194 distinct eyes (98 patients in total)^26^. The average age of the subjects is 66.9 years with a standard deviation of 9.7 years; 66% are female^27^. All subjects have some form of OAG with average and median VF sensitivities across all eyes/visits of 23 and 27 decibels, respectively (standard deviation: 10; range: 0–49)^27^. Three of the eyes were removed from the final analysis because of missing information on the clinical determination of progression. The mean follow-up time for the remaining participants is 2.6 years (range: 0.2–9.4) with an average of 7.4 tests (range: 2–21) per eye. Glaucomatous VF progression status was determined at the final visit based on the expertise of two independent clinicians. A third clinician evaluated any discrepancies between the two primary assessors (only necessary for 13 VF series). The clinicians had access to GPA output and VF printouts of the tests across time when making their determinations. In total, there are 141 (74%) stable and 50 (26%) progressing eyes. Informed consent was obtained from each participant in the study. The trial adhered to the tenets of The Declaration of Helsinki and was approved by the University of Western Australia’s ethics committee. Full information on the VF data collection process has been previously described^26,27^.

### Spatiotemporal boundary detection (STBound)

The statistical method developed in Berchuck *et al*. provides a novel approach to diagnosing glaucomatous VF progression^25^. Unlike previous methods that focus on estimating trends in changing VF sensitivities over time, it quantifies changes in the spatial correlation structure of VF data across time and uses the developed metric, STBound, to detect VF progression.

For each VF test, a statistical method known as spatial boundary detection with a dissimilarity metric is applied to the collected data and the spatial correlation structure of the data at the current visit is estimated. This correlation is partially determined by the fact that in glaucomatous optic nerve damage, VF damage occurs in certain characteristic patterns determined by the anatomy of the retinal ganglion cells and their axonal pathways as they bundle together to form the optic nerve^28^. If the estimated spatial correlation is changing substantially over time, we showed that this is highly predictive of progression. If the estimates of spatial correlation are stable across time, the eye is more likely to be classified as non-progressing. In order to quantify the variability in spatial correlation across time and to create a standardized metric to facilitate comparisons across eyes, we used the coefficient of variation (CV) (large value: high variability; small value: low variability).

While we previously only investigated the estimated CV as a predictor of VF progression, in this study we explore the use of the estimated CV, its standard error, and their interaction in combination, and refer to this set of predictors as STBound. By including these additional predictors in the framework, this work extends the predictive capabilities of the original work and yields a more clinically useful metric for monitoring VF progression. Supplementary Fig. S1 shows an example of the variability in the estimated spatial correlation across time for two patients in the study, one progressing, one stable, along with the individual components of the STBound metric and predicted probabilities of progression based on STBound. The full details on the statistical model, including model fitting, can be found elsewhere^25^. The model is freely available and implemented in the R package womblR^29^.

### Model comparison

In this study, we further explored the potential of STBound in diagnosing VF progression in comparison to existing methods and its ability to correctly detect disease worsening earlier than these methods. To formally make these comparisons, we constructed logistic regression models, regressing all developed predictors of VF progression on the clinical assessment of progression and calculated the Akaike Information Criterion (AIC), area under the receiver operating characteristic (ROC) curve (AUC) (full and partial), sensitivity, and specificity. The final metrics used for diagnosing progression are the predicted probabilities of progression from each of the fitted logistic regression models.

We compared STBound to a global index regression method (GI), point-wise linear regression (PLR)^16^, the mean regression slope method (MS)^30^, permutation of pointwise linear regression (PoPLR)^31^, and Spatial PROGgression (SPROG)^26^. In GI, we first averaged the VF sensitivities during an exam to produce a single VF value. We then repeated this process separately for data from each VF test. Finally, we regressed these averages (one from each test) on time to determine the average rate of change in the VF across time. Any alternative global indices (e.g., mean deviation) could be used in the regression and we chose the average VF sensitivities due to ease of interpretation and availability of information. We used the estimated regression slope and p-value, along with their interaction, as predictors of progression. Including the p-value when determining progression allows us to differentiate between statistically significant and non-significant slope estimates and should improve our ability to detect true progression.

GI will work well when large sections of the VF are degrading over time, but may struggle to detect highly localized damage^32–34^. As a result, we also considered several location-specific analyses. PLR is a commonly used trend-based point-wise method, in which detection of progression corresponds to statistically significant slopes of the VF location-specific regression lines (sensitivities regressed on time)^16^. We present results from four versions of PLR, where the progression criterion is based on the 1^st^, 2^nd,^ 3^rd^, and 4^th^ smallest p-values from all location-specific regressions being less than 0.001 (P1, P2, P3, P4)^16^. Therefore, the 1^st^, 2^nd^, 3^rd^, and 4^th^ smallest p-values are used as predictors of progression in the logistic regression analyses. In MS^30^, we first computed PLR at each VF location and then averaged the estimated slopes whose p-values were < 0.01. This average slope value was used as a predictor of progression in the logistic regression analysis. PoPLR^31^ represents an advanced version of PLR that creates a test statistic defined as the sum of the negative logged PLR p-values that were less than 0.05. A larger value is indicative of progression. This test statistic was used as a predictor of progression in the logistic regression analysis.

We also include an advanced spatiotemporal statistical method for comparison^26^. SPROG simultaneously models sensitivities from each VF location linearly over time using hierarchical Bayesian disease mapping techniques while also accounting for the underlying structural-functional correlates of the eye. If the overall slope that describes change in sensitivities over time is statistically significantly less than zero, then SPROG classifies the eye as progressing. We included the posterior probability that the global slope is less than zero as a predictor of progression in the logistic regression analysis. Full details of SPROG have been previously described^26^. A full summary of the progression metrics is given in Table 1.

**Table 1:** Summary of criteria included in each diagnostic method.

Method	Progression Metric
GI	Slope and corresponding p-value from regressing the average (across all VF locations) differential light sensitivity (DLS) from each test across time, and their interaction^29^.
MS	Overall mean slope (MS) of DLS over time for all location specific regression lines with a p-value < 0.01^29^.
P1	1^st^ order statistic of slope p-values from the location specific regression^16^.
P2	2^nd^ order statistic of slope p-values from the location specific regression^16^.
P3	3^rd^ order statistic of slope p-values from the location specific regression^16^.
P4	4^th^ order statistic of slope p-values from the location specific regression^16^.
PoPLR	Overall p-value for the negative log sum of the slope p-values from the location specific regressions less than 0.05 based on permutation^31^.
SPROG	The estimated overall slope that describes change in sensitivities over time across the entire VF after fitting a hierarchical Bayesian disease mapping statistical model^26^.
STBound	The posterior mean and standard deviation of the coefficient of variation of the parameter that describes the spatial correlation of the VF data, and their interaction^25^.

For each of the metrics, the covariates that are listed are included in a logistic regression model used to predict clinical assessment of progression. The resulting predicted probabilities of progression are used as the progression metric.

We compared metrics using AIC, AUC, partial AUC (pAUC), sensitivity, and specificity. A smaller value of AIC indicates an improved model fit, while larger values of AUC, pAUC, sensitivity, and specificity indicate superior discriminatory ability^35,36^. Based on previous work in this area, we limited the pAUC to regions of clinically relevant specificity, 85–100%^20^. In addition, we used Pearson correlation and statistical hypothesis tests to investigate the similarity between each of the considered metrics. Methods that yield predictors with low correlation may be combined in order to improve predictive performance.

### Assessing early stage progression

Diagnosing glaucomatous VF progression early is critical for limiting irreversible vision loss, thus we study the performance of each metric in the early phases. To accomplish this, we study the longitudinal trends in AUC, pAUC, sensitivity, and specificity for each metric in the initial years from baseline with respect to the clinical determination of progression on the final testing date. We estimate these summaries at half-year increments up to 4.5 years of follow-up. Only visits that occurred on or before the cutoff time are included in each analysis. This imitates a clinical setting where each metric is calculated at every visit and progression is diagnosed.

## Results

### Diagnostic capability of the metrics

The results comparing the diagnostic capabilities of the different methods at the end of the study period are shown in Table 2. Assessment of the performance of each individual metric (“Without GI” columns of Table 2) indicates that each of the trend-based methods are highly predictive of progression, as noted by the significant marginal p-values that describe the overall utility of the predictor. The global metric, GI (AIC: 204.11; AUC: 0.70; pAUC: 0.26), is superior to the majority of existing trend-based methods (although not statistically significant, p-values 0.05–0.99) in terms of AIC (210.46–217.55), AUC (0.63–0.69), and pAUC (0.13–0.32). The only exception is the performance of PoPLR with respect to pAUC (0.32 vs. 0.26). The newly developed metric, STBound, has improved diagnostic ability compared to the competing trend-based methods, including GI, with respect to AIC (197.77) and AUC (0.74). It also performs similarly to PoPLR with respect to pAUC (0.31 vs. 0.32); though the AUC and pAUC values are not statistically significantly larger than the values produced by GI (p-values: 0.51, 0.60, respectively). However, this improved performance indicates that STBound may be useful for diagnosing progression on its own.

**Table 2:** Assessing the diagnostic capability of the metrics. Each metric is regressed against the clinical assessment of progression, both with and without the global index (GI).

Metric	Without GI				With GI			
	AIC	AUC	pAUC	P^*^	AIC	AUC	pAUC	P^†^
GI	204.11	0.70	0.26	<0.001	—	—	—	—
MS	210.46	0.64	0.24	<0.001	204.49	0.70	0.31	0.203
P1	214.16	0.64	0.13	0.002	201.50	0.74	0.28	0.032
P2	217.55	0.64	0.26	0.014	204.52	0.71	0.26	0.207
P3	215.13	0.63	0.19	0.004	203.43	0.72	0.27	0.102
P4	214.12	0.65	0.18	0.002	203.32	0.71	0.28	0.095
PoPLR	212.94	0.69	0.32	0.001	206.10	0.70	0.26	0.906
SPROG	214.84	0.67	0.15	0.003	206.11	0.70	0.26	0.953
STBound	197.77	0.74	0.31	<0.001	180.41	**0.81**	**0.45**	<0.001

To compare models, AIC, AUC and pAUC are reported. The pAUC is limited to the clinically relevant range of specificity, 85–100%. Also reported are p-values corresponding to hypothesis tests ^*^for each metric marginally, and ^†^in addition to GI. The bold cell indicates a significant improvement in AUC or pAUC over the GI model at the α = 0.05 level of significance.

### Correlation between methods

The major strength of STBound is the fact that it does not rely on an underlying trend-based method and is therefore providing unique predictive information to the decision-making process. This fact is shown clearly in Table 3 where the Pearson correlation estimates and corresponding p-values between each of the fitted probabilities of progression based on the metrics from Table 2 are presented. The estimated correlations are presented below the diagonal with the p-values above the diagonal. All of the existing trend-based methods are highly correlated with each other (all p-values ≤ 0.05). STBound, however, is uncorrelated with the existing methods as indicated by the large bolded p-values (all p-values > 0.05). These correlations suggest that STBound explains a unique alternative pathway in understanding glaucomatous VF progression that is not incorporated into existing trend-based methods. As such, STBound can be used in conjunction with trend-based methods.

**Table 3:** Correlation matrix and hypothesis test results for the diagnostic metrics.

Metric	GI	MS	P1	P2	P3	P4	PoPLR	SPROG	STBound
GI	—	<0.01	<0.01	<0.01	<0.01	<0.01	<0.01	<0.01	**0.26**
MS	0.55	—	<0.01	<0.01	<0.01	<0.01	<0.01	<0.01	**0.93**
P1	0.26	0.34	—	<0.01	<0.01	<0.01	<0.01	0.05	**0.48**
P2	0.31	0.25	0.83	—	<0.01	<0.01	<0.01	<0.01	**0.47**
P3	0.33	0.21	0.72	0.92	—	<0.01	<0.01	<0.01	**0.23**
P4	0.38	0.24	0.68	0.85	0.92	—	<0.01	<0.01	**0.18**
PoPLR	0.67	0.43	0.35	0.43	0.45	0.50	—	<0.01	**0.15**
SPROG	0.57	0.32	0.14	0.27	0.26	0.28	0.81	—	**0.11**
STBound	**0.08**	**−0.01**	**0.05**	**0.05**	**0.09**	**0.10**	**0.10**	**0.11**	—

The estimated Pearson correlations and p-values between the metrics are presented below and above the diagonal, respectively. Bold values indicate that the correlation does *not* statistically differ from zero at the *α* = 0.05 level of significance.

### Combination of methods

We tested the performance of combining GI with the other metrics, including STBound, relative to these metrics alone. GI was selected because it was shown to be the top performing trend-based method in the study overall. The metrics were combined by including the predictor(s) corresponding to a selected method into a single logistic regression model that also included the predictors from the GI model (i.e., estimated regression slope, p-value, and interaction). This allowed us to determine if any of the metrics offered additional information with respect to detecting progression outside of that already provided by GI. The “With GI” columns of Table 2 display the results when each of the metrics are combined with GI. Based on the correlation analysis in Table 3, we did not expect large improvements in diagnostic ability when GI is added to the other trend-based methods due to their large degree of similarity. This appears to be the case as p-values that describe the statistical significance of the added metrics are generally >0.05, with the exception of P1 (p-value = 0.032) (though the improvements in AUC and pAUC are not statistically significant). STBound is the only metric that improves the predictive performance across all diagnostic indicators. When STBound is added to GI, the increase in AUC and pAUC are statistically significant with p-values of 0.002 and 0.003, respectively. The model with GI and STBound (referred to as GI + STBound) has optimal characteristics with a minimum AIC of 180.41 vs. 197.77, and maximum AUC and pAUC of 0.81 vs. 0.74 and 0.45 vs. 0.31, respectively. The estimated regression coefficients, standard errors, and p-values for GI + STBound are presented in Supplementary Table S1. When PoPLR is added to GI, the pAUC actually decreases, which may be indicative of the redundancy in the information that both provide.

**Figure 1:**
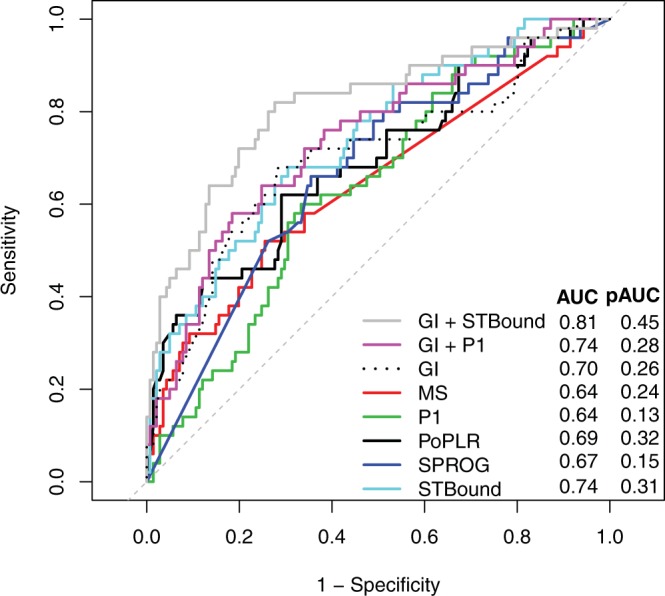
Receiver operating characteristic (ROC) curves for statistically significant diagnostic metrics.

In Fig. 1, ROC curves are displayed for each of the metrics from Table 2 that were significant, GI, MS, P1, PoPLR, SPROG, STBound, GI + P1, and GI + STBound. We only present P1 to represent the PLR methods in order to simplify the figure. The ROC curve can be interpreted as sensitivity and (1-specificity) over various thresholds. Figure 1 confirms the results in Table 3, as the ROC curve of GI + STBound suggests significant improvement over the STBound curve, in particular in the region of high specificity. Although P1 was shown to improve GI, it is clear from the ROC curves that the improvement is marginal at best.

### Determination of progression over time

**Figure 2:**
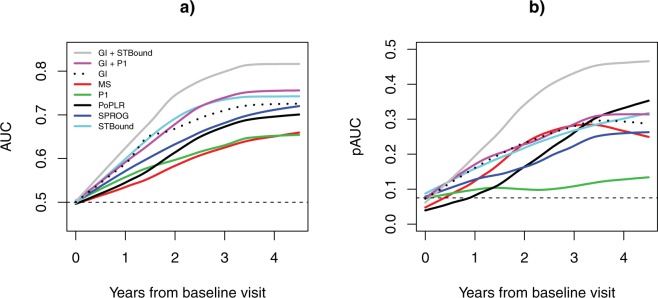
Performance of diagnostic metrics in the initial years from baseline visit, AUC (**A**) and pAUC (**B**). Estimates are presented as smooth curves using the LOESS method. The horizontal dashed line indicates the value at which a metric does not contribute any diagnostic information.

We also explored the performance of the metrics in the early years of the study with respect to progression determined at the end of the study. The presentation of the longitudinal trends of AUC and pAUC are displayed in Fig. 2 for all metrics found to be significant in Table 2 (again only including P1 to represent the PLR methods). The figure presents smoothed local regression estimates of AUC and pAUC^37^. In Fig. 2A, the AUC trends are presented, where GI + STBound has superior AUC over the entire time period. The pAUC trends are displayed in Fig. 2B, with an even more pronounced separation of GI + STBound over the other metrics. Supplementary Table S2 presents the raw estimates of AUC and pAUC at the first four years of the study, corresponding to the smoothed lines presented in Fig. 2. Bolded entries in the table coincide to values that significantly improve upon the GI value at the same year (${\rm{\alpha }}$ = 0.05 level of significance), demonstrating that the gains seen in GI + STBound in Fig. 2 are statistically significant. Overall, GI + STBound is shown to produce improved diagnostic metrics earlier than the competing methods.

**Figure 3:**
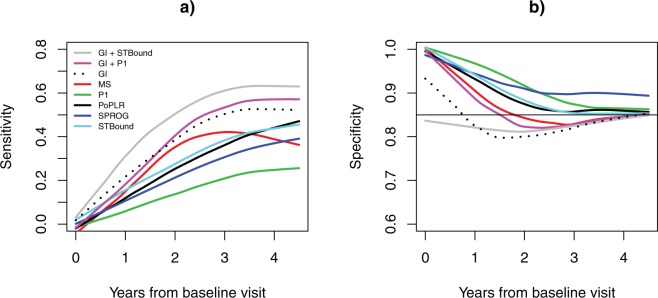
Demonstrating the performance of sensitivity (**A**) and specificity (**B**) in the initial years from baseline visit. Both sensitivity and specificity are obtained by using a clinically motivated threshold defined to maximize sensitivity, while forcing the specificity to be no smaller than 85%. Estimates are presented as smooth curves using the LOESS method.

Figure 3 and Supplementary Table S3 present sensitivity and specificity estimates for each metric annually for four years after the baseline visit and at the end of the study. The sensitivity and specificity values are obtained through calculation of an optimal threshold. The threshold in use is motivated clinically, so that the specificity is guaranteed to be at least 85%^20^, while maximizing sensitivity. The thresholds are calculated using the completed study and are given in Supplementary Table S3. In Fig. 3, it is clear that the sensitivity is superior in the GI + STBound model beginning early in follow-up (0.34–0.64 vs. 0.06–0.60). Due to the definition of the threshold, the specificity for all models is around the 85% level throughout follow-up. Specifically in Supplementary Table S3, by the end of the study GI + STBound has optimal operating characteristics, with specificity in the clinical range, while showing statistically significant improvement in sensitivity over GI. In the earlier years, the specificity does not deteriorate, and the sensitivity only begins to drop-off significantly within two years from baseline, with sensitivity of 0.52.

**Figure 4:**
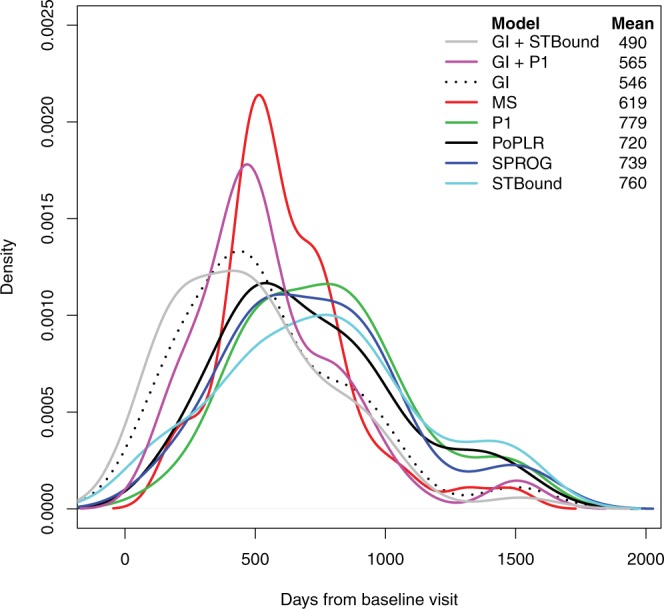
Density estimates of the time of first diagnosis for each model in days from baseline visit along with the mean number of days.

Finally, we explore how much faster the GI + STBound metric is able to successfully diagnose progression in terms of days from baseline visit. For all 50 progressing patients, we determined which visit each metric would have identified the patient as progressing using the clinically motivated thresholds in Supplementary Table S3. If a model was incapable of detecting progression at the end of the study, those patients were assigned the date of the last visit. Figure 4 presents the density estimate of the time of first diagnosis for each model in days from baseline visit along with the mean number of days. The GI + STBound metric is capable of diagnosing progressing earliest, improving approximately two months over the second fastest, GI.

## Discussion

This study validates the method introduced previously as a modeling framework for diagnosing glaucomatous VF progression^25^. The method utilizes an innovative spatiotemporal boundary detection framework that accounts for the characteristic patterns of visual loss in the setting of glaucoma, resulting in a novel diagnostic metric that represents a departure from standard trend-based methods. Instead, STBound quantifies change in the spatial correlation of the VF data, representative of underlying damage to the optic disc across time.

When establishing a novel diagnostic testing algorithm, assessing its predictive performance in isolation is fundamental; however, demonstrating the incremental performance over established metrics is necessary to demonstrate its clinical utility. All of the metrics in the study were found to be marginally statistically significant predictors of progression (Table 2, “Without GI” columns). This was not surprising, since all but STBound were established diagnostic methods used for the detection of glaucoma progression. However, only STBound provided improved performance in addition to GI (Table 2, “With GI” columns). Although P1 was also significant, it is clear from the results that this improvement was minimal. The established metrics GI, PLR, MS, PoPLR, and SPROG are derived from analyzing changes in the mean trend of VF sensitivities across time and therefore, explain similar components of progression. This is confirmed in Table 3, where the Pearson correlation estimates for all of the mean trend metrics are significant. On the other hand, the Pearson correlations between STBound and the mean trend metrics are close to zero, establishing their independence. As an independent diagnostic, STBound has clinical utility in its ability to assess an adjacent pathway in glaucoma progression.

Having demonstrated the utility of STBound clinically, we then established it as significantly superior in the important early stages from baseline visit as diagnosing progression early is critical for limiting irreversible vision loss. The AUC, pAUC, sensitivity, and specificity show GI + STBound is superior over the entire early stage (Figs 2, 3; Supplementary Tables S2, S3). This indicates the accelerated efficacy of STBound in the early stages from baseline. It also indicates the independence of STBound from GI, as GI + STBound is able to improve over the GI curve, while the GI + P1 metric makes no notable improvements. Early detection of glaucoma progression is important clinically as interventions can be effective in reducing a patient’s likelihood of vision loss^38^. Therefore, diagnostic metrics such as STBound are critical for providing improved patient outcomes.

This study has a few limitations. First, the final determination of VF progression was based on agreement among a group of expert clinicians who had access to GPA output and raw VF data. However, while no widely accepted reference standard currently exists to define VF progression, many researchers agree that assessment of a glaucoma patient’s data and agreement among a group of clinical experts is currently the most acceptable standard definition when introducing new analytic models^39^. We also note that only 13 out of the 191 assessed VF series resulted in disagreement among the clinicians, likely indicating clear progression/non-progression outcomes. Past statistical methods work have also used this outcome when creating, validating, and comparing new and existing methods, including with the same dataset used in this analysis^19,23,26^. We also did not have access to pattern standard deviation (PSD) and/or loss variance (LV) during each VF test, nor did we have the exact ages of the subjects at the time of testing needed to directly calculate these metrics ourselves. In order to preserve patient confidentiality, we only received de-identified raw data from the original study. These metrics likely describe VF loss differently than the trend-based methods and could have been added to the set of competing methods. Future work should compare the performance of STBound to metrics like PSD and LV that quantify the variability in local vision loss, similar to STBound. In addition, future work is also needed to determine if certain aspects of STBound might be able to differentiate people with and without glaucoma, allowing for STBound to be used during each stage of disease management. Finally, the inclusion of structural indicators of disease progression into our regression framework could result in an improved ability to differentiate true progression from random variability, leading to preservation of a patient’s remaining vision.

With further validation, we propose that the metric STBound be integrated into the clinical environment. STBound requires a minimum of two visits to compute and from Fig. 4, we know that it can be used to decrease the time until a correct progression diagnosis by two months on average over GI. In practice, clinicians can implement the method defined in this study based on the clinically defined thresholds in Supplementary Table S3. We suggest using the GI + STBound model, as it has optimal operating characteristics across the entire study (Supplementary Table S3). These characteristics are relatively conservative, and this is due to the definition of the threshold, which prioritizes high values of specificity. We note that this threshold can be adjusted to accommodate different sensitivity/specificity preferences. GI + STBound can be calculated using the regression coefficients presented in Supplementary Table S1 along with the individual components of STBound that can be obtained using the womblR^29^ R package.

## Supplementary information

Supplementary Information

## Data Availability

The deidentified dataset that includes each subject’s clinical determination of progression and all calculated progression metrics (including across time) is available from the corresponding author on request.
