# Supplementary material for: Improved Detection of Visual Field Progression Using a Spatiotemporal Boundary Detection Method
Source: Sci Rep. 2019 Mar 15;9:4642. doi: 10.1038/s41598-018-37127-z (PMC6420602; doi:10.1038/s41598-018-37127-z)
Supplement: Supplementary file 1 — Supplementary Information [file 41598_2018_37127_MOESM1_ESM.pdf]

Supplementary Information

Title
Improved Detection of Visual Field Progression Using a Spatiotemporal Boundary Detection Method

Authors
Samuel I. Berchuck,[1] Jean-Claude Mwanza,[2] Angelo P. Tanna,[3] Donald L. Budenz,[2] and Joshua L. Warren[4,*]

Affiliations
[1]Department of Biostatistics, University of North Carolina-Chapel Hill, NC, USA
[2]Department of Ophthalmology, University of North Carolina-Chapel Hill, NC, USA
[3]Department of Ophthalmology, Northwestern University, Illinois, USA
[4]Department of Biostatistics, Yale University, Connecticut, USA
*joshua.warren@yale.edu

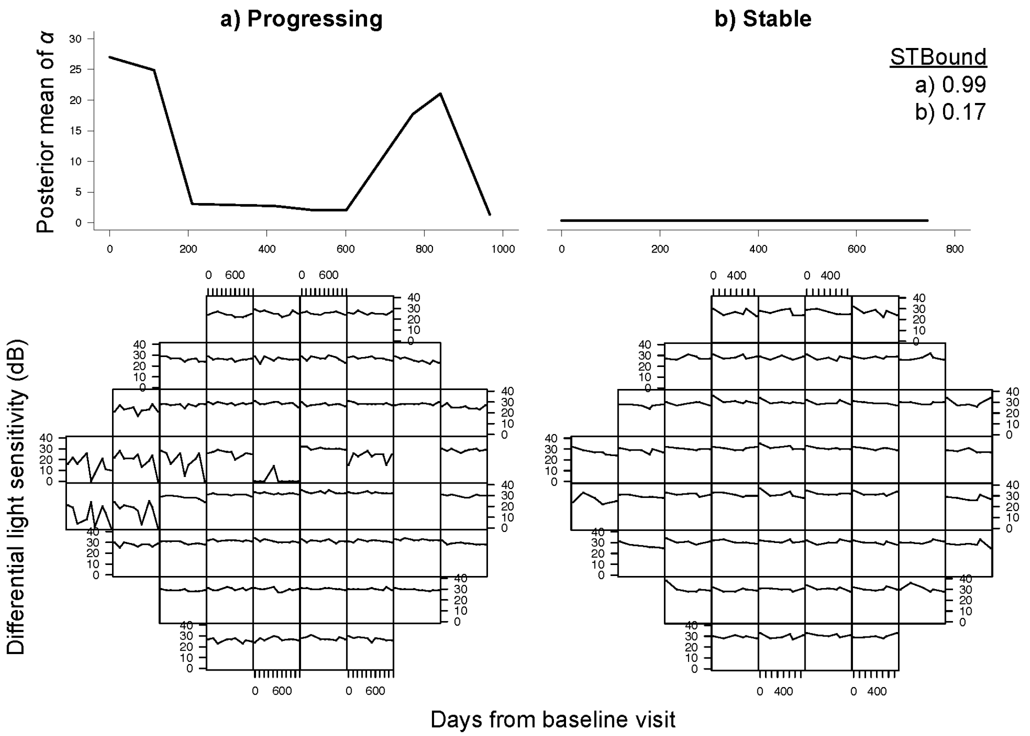

Supplementary Figure S1. Longitudinal series of visual fields (VFs) for one progressing (left) and stable (right) eye are presented in the bottom row. Each cell represents the observed VF sensitivities over all visits. The progressing eye has more variability across the VF and in particular in the region between the superior and inferior sectors. In the top row, the estimated parameter that describes the spatial correlation in the VF data is plotted over time and the corresponding predicted probabilities of progression based on values of STBound are given as 0.99 and 0.17 for the progressing and stable eye, respectively. For these two patients STBound indicates that eye a) is much more likely to be progressing.

Supplementary Table S1. Estimated coefficients from the logistic regression model combining global index (GI) and STBound.

Variable	Estimate (SE)	P-value
**Intercept**	-1.75 (0.97)	0.073
**GI: P-value**	-0.25 (0.75)	0.738
**GI: Slope**	-13.73 (4.20)	0.001
**GI: P-value * Slope**	18.7 (2.83)	0.008
**STBound: Mean**	7.47 (6.53)	0.172
**STBound: SE**	-8.92 (7.37)	0.011
**STBound: Mean * SE**	-13.37 (14.97)	0.372

Supplementary Table S2. Estimates of AUC and pAUC presented at yearly intervals.

Metric		Years from Baseline Visit				
		1	2	3	4	End
**GI**	AUC	0.55	0.66	0.73	0.73	0.70
	pAUC	0.07	0.23	0.31	0.30	0.26
**MS**	AUC	0.52	0.58	0.64	0.66	0.64
	pAUC	0.07	0.22	0.29	0.29	0.24
**P1**	AUC	**0.56**	0.60	0.65	0.65	0.64
	pAUC	0.12	0.10	0.11	0.14	0.13
**PoPLR**	AUC	0.53	0.62	0.69	0.70	0.69
	pAUC	0.03	0.14	0.31	0.34	0.32
**SPROG**	AUC	0.58	0.63	0.71	0.71	0.67
	pAUC	0.11	0.14	0.27	0.26	0.15
**STBound**	AUC	0.59	0.70	0.74	0.75	0.74
	pAUC	0.16	0.22	0.27	0.32	0.31
**GI + P1**	AUC	0.57	0.69	0.76	0.75	0.74
	pAUC	0.20	0.21	0.32	0.32	0.28
**GI + STBound**	AUC	0.52	0.74	**0.82**	**0.82**	**0.81**
	pAUC	0.14	**0.35**	**0.45**	**0.47**	**0.45**

These raw estimates correspond to the LOESS smoothed curves presented in Fig. 2. Bold cells indicate a significant improvement in either AUC or pAUC over the corresponding values of the global index (GI) at the $\alpha = 0.05$ level of significance.

Supplementary Table S3. Sensitivity (sens.) and specificity (spec.) for each metric using the clinical definition for thresholds.

Metric	Threshold		Years from Baseline Visit				
			1	2	3	4	End
**GI**	0.344	Sens.	0.22	0.38	0.54	0.52	0.48
		Spec.	0.78	0.76	0.82	0.84	0.85
**MS**	0.322	Sens.	0.12	0.38	0.42	0.42	0.34
		Spec.	**0.88**	0.79	0.83	0.85	0.85
**P1**	0.338	Sens.	0.06	0.16	0.22	0.26	0.24
		Spec.	**0.90**	**0.91**	0.86	0.87	0.85
**PoPLR**	0.353	Sens.	0.12	0.24	0.40	0.46	0.44
		Spec.	**0.85**	**0.85**	0.86	0.87	0.85
**SPROG**	0.323	Sens.	0.10	0.22	0.36	0.38	0.52
		Spec.	**0.94**	**0.88**	**0.91**	**0.89**	0.74
**STBound**	0.302	Sens.	0.16	0.28	0.40	0.46	0.44
		Spec.	**0.94**	**0.87**	0.85	0.86	0.85
**GI + P1**	0.335	Sens.	0.18	0.42	0.60	0.58	0.52
		Spec.	0.81	0.79	0.83	0.84	0.85
**GI + STBound**	0.325	Sens.	**0.34**	**0.52**	0.64	0.64	**0.64**
		Spec.	0.77	0.81	0.83	0.84	0.85

These raw estimates correspond to the LOESS smoothed curves presented in Fig. 3. The bold cells indicate a significant improvement in either sens. or spec. from the corresponding values of the global index (GI) at the $\alpha = 0.05$ level of significance.
